# Maternal obesity and the risk of neonatal sepsis: a meta-analysis

**DOI:** 10.3389/fped.2026.1886925

**Published:** 2026-07-31

**Authors:** Kaiting Yang, Hong Yang, Chuanfeng Li

**Affiliations:** 1 Department of Neonatology, Nanyuan Campus, Maternal and Child Health Hospital of Qujing, Qujing, China; 2 Department of Neonatology, Liaokuo Campus, Maternal and Child Health Hospital of Qujing, Qujing, China

**Keywords:** meta-analysis, neonatal sepsis, obesity, pregnancy, risk factor

## Abstract

**Background:**

Maternal obesity is increasingly prevalent worldwide and has been associated with adverse neonatal outcomes. However, the association between maternal obesity and the risk of neonatal sepsis remains uncertain. This meta-analysis aimed to evaluate the association between maternal obesity before or during early pregnancy and the risk of neonatal sepsis in offspring.

**Methods:**

PubMed, Embase, and Web of Science were searched for observational studies evaluating the association between maternal obesity and neonatal sepsis. Maternal obesity was generally defined as prepregnancy or early-pregnancy body mass index (BMI) ≥30 kg/m^2^. Risk ratios (RRs) and 95% confidence intervals (CIs) were pooled using a random-effects model accounting for potential heterogeneity.

**Results:**

Nine observational studies involving 2,146,883 women were included. Pooled analysis showed that maternal obesity was associated with a significantly increased risk of neonatal sepsis (RR: 1.46, 95% CI: 1.15–1.85, *p* = 0.002; I^2^ = 58%). Stratified analyses according to maternal obesity severity showed progressively increased RRs for neonatal sepsis among women with BMI values of 30–34.9, 35–39.9, and ≥40 kg/m^2^ (RRs: 1.34, 1.87, and 2.26, respectively; *p* for subgroup difference = 0.12). The association was significant in general obstetric populations but not in studies restricted to preterm delivery. Stronger associations were observed in studies reporting early-onset sepsis than overall neonatal sepsis. Meta-regression analysis showed that mean gestational age at delivery and neonatal birthweight significantly influenced the pooled effect size.

**Conclusions:**

Maternal obesity before or during early pregnancy is associated with an increased risk of neonatal sepsis in offspring.

**Systematic Review Registration:**

PROSPERO: CRD420261400345.

## Introduction

Neonatal sepsis is a major cause of neonatal morbidity and mortality worldwide despite advances in perinatal and neonatal care (1, 2). It is characterized by systemic infection occurring during the neonatal period and is generally classified as early-onset sepsis (EOS), which typically occurs within the first 72 h after birth, or late-onset sepsis (LOS), which develops later during the neonatal period (3). Neonatal sepsis is associated with prolonged hospitalization, increased healthcare burden, neurodevelopmental impairment, and increased risk of death, particularly among preterm and low-birth-weight infants (4). Although several maternal and neonatal risk factors for neonatal sepsis have been established, including prematurity, prolonged rupture of membranes, chorioamnionitis, invasive procedures, and low birth weight, the incidence and burden of neonatal sepsis remain substantial globally (5, 6). Therefore, identifying potentially modifiable maternal risk factors associated with neonatal sepsis is important for improving risk stratification and preventive strategies during pregnancy and the perinatal period.

Maternal obesity, commonly defined as body mass index (BMI) ≥30 kg/m^2^, has become increasingly prevalent among women of reproductive age worldwide and represents a major public health concern (7, 8). In obstetric research, prepregnancy or early-pregnancy BMI is generally considered more reliable and clinically meaningful than BMI measured later during pregnancy because it is less affected by gestational weight gain and pregnancy-related physiological changes (9, 10). Maternal obesity before or during early pregnancy has been associated with multiple adverse maternal and neonatal outcomes, including gestational diabetes, hypertensive disorders of pregnancy, cesarean delivery, fetal macrosomia, preterm birth, and neonatal intensive care unit admission (11, 12). Several biological mechanisms may potentially link maternal obesity to neonatal sepsis, including chronic low-grade maternal inflammation, altered maternal and placental immune responses, dysregulated maternal microbiota, increased risk of obstetric complications, and impaired neonatal immune adaptation (13, 14). In recent years, a growing number of observational studies have investigated the association between maternal obesity and neonatal sepsis (15, 16). However, the reported findings remain inconsistent, and the magnitude of the association is uncertain (17–25). Moreover, differences in study populations, definitions of maternal obesity, and diagnostic methods for neonatal sepsis may contribute to heterogeneity across studies (17–25). Therefore, this meta-analysis was conducted to comprehensively evaluate the association between maternal obesity before or during early pregnancy and the risk of neonatal sepsis in offspring.

## Methods

The meta-analysis was carried out in accordance with established methodological guidance, following the principles outlined in the PRISMA 2020 statement (26) and the Cochrane Handbook for Systematic Reviews of Interventions (26), encompassing protocol planning, study selection, data collection, statistical analysis, and results interpretation. The study protocol was registered prospectively in the PROSPERO database (registration number: CRD420261400345).

### Database search

A systematic literature search was conducted in PubMed, Embase, and Web of Science to identify studies that met the eligibility criteria for inclusion. The search strategy was constructed using the combination of the following terms (1): “maternal obesity” OR obesity OR obese OR overweight OR “body mass index” OR BMI OR “maternal BMI” OR “prepregnancy obesity” OR “pre-pregnancy obesity” OR “early pregnancy obesity”; and (2) “neonatal sepsis” OR sepsis OR “early-onset sepsis” OR EOS OR “late-onset sepsis” OR LOS OR “neonatal infection” OR “neonatal bacterial sepsis”. Only full-text articles published in English involving human participants were eligible for inclusion. Additionally, the reference lists of relevant reviews and original studies were manually examined to identify further potentially eligible publications. All databases were searched from their inception up to March 12, 2026. Detailed search strategies for each database are presented in Supplementary File S1.

### Study inclusion and exclusion criteria

The selection of studies was guided by the PICOS principle:

#### P (population)

Studies including pregnant women and their offspring (neonates, typically ≤28 days of life) from population-based or hospital-based settings.

#### I (exposure)

Maternal obesity, defined as prepregnancy or early-pregnancy BMI ≥30 kg/m^2^, reported as a categorical variable. In general, early-pregnancy BMI refers to BMI measured during early gestation, typically at the first prenatal visit within the first trimester, and is commonly used as a proxy for prepregnancy BMI in observational studies.

#### C (comparator)

Non-obese mothers, defined as those with normal BMI (18.5–24.9 kg/m^2^).

#### Outcome (O)

Neonatal sepsis, including EOS and/or LOS, defined by clinical diagnosis, laboratory confirmation, International Classification of Disease (ICD) codes, or clearly reported diagnostic criteria. When available, EOS and LOS were extracted and analyzed separately; otherwise, a composite outcome of overall neonatal sepsis was used.

#### S (study design)

Observational studies with longitudinal follow-up, including prospective or retrospective cohort studies, nested case-control studies, or *post-hoc* analyses of clinical trials.

Studies were excluded if they: (1) did not report BMI-defined maternal obesity as a categorical exposure (e.g., reported only continuous BMI without categorizable data); (2) evaluated other exposures such as gestational weight gain, diabetes, or metabolic syndrome without separable BMI-based obesity data; (3) did not report neonatal sepsis or provided insufficient information to define the outcome (including unclear diagnostic criteria); (4) were non-original studies (reviews, editorials), case reports, or animal studies; (5) involved overlapping populations, in which case only the study with the largest sample size was included; or (6) lacked sufficient data to derive effect estimates and corresponding confidence intervals.

### Study quality assessment

Two reviewers independently performed the literature search, screened studies for eligibility, extracted data, and assessed study quality. Any disagreements were resolved through discussion, and when necessary, a third investigator was consulted to reach consensus. The methodological quality of the included studies was evaluated using the Newcastle–Ottawa Scale (NOS) (27), which assesses study quality across three domains: selection, comparability, and outcome assessment. NOS scores range from 1 to 9, with studies scoring ≥ 8 considered to be of high quality.

### Data collection

Data extraction was conducted independently by two reviewers using a standardized data collection form. Extracted variables included study characteristics (first author, publication year, study design, and country), participant characteristics (source of women, number of women included, mean maternal age, mean gestational age [GA] at delivery), timing of BMI evaluation, cutoffs of BMI for defining maternal obesity, number of neonates born to women with obesity, mean birth weight, and sex distribution of the neonates, methods for the diagnosis of neonatal sepsis, number of neonatal sepsis cases, and variables adjusted for in the analyses examining the association between maternal obesity and incidence of neonatal sepsis.

### Statistical analyses

The association between maternal obesity and the risk of neonatal sepsis was evaluated by pooling risk ratios (RRs) and their corresponding 95% confidence intervals (CIs) (26), comparing women with and without obesity before or during early pregnancy. If data from multiple models were available, those from the most adequately adjusted model were extracted for subsequent analysis. When necessary, effect estimates and their standard errors were derived from the reported 95% CIs or *p* values. Before pooling, all effect measures were converted to the natural logarithmic scale to improve normality and ensure variance stabilization for meta-analysis (26). To evaluate variability across studies, we applied the Cochrane Q test and calculated the I² statistic (28). Heterogeneity was categorized based on I² values as low (< 25%), moderate (25%–75%), or high (> 75%). Pooled effect estimates were calculated using the inverse variance (IV) approach within a random-effects framework (DerSimonian–Laird method) to account for potential between-study variability (26). Sensitivity analyses were performed using a leave-one-out approach, in which each study was sequentially excluded to assess the stability and robustness of the pooled results (29). For studies reporting multiple datasets according to maternal obesity severity, stratified analyses were performed to explore the potential dose-response relationship between maternal obesity severity and the risk of neonatal sepsis. To identify possible sources of between-study variability, we performed predefined subgroup analyses stratified by mean maternal age, source of the women (preterm delivery only vs. overall obstetric population), methods for diagnosis of neonatal sepsis (culture-proven vs. diagnosis from medical chart or ICD codes), definition of neonatal sepsis (EOS vs. overall sepsis), analytic model (univariate vs. multivariate), and study quality scores. Medians of continuous variables were selected as the cutoffs for defining the subgroups in order to balance the number of available studies in each subgroup. Additionally, univariate meta-regression analyses were conducted to examine the potential impact of study-level characteristics on the association between maternal obesity and the risk of neonatal sepsis, such as mean maternal age, mean GA at delivery, mean birthweight of neonates born to women with obesity, and NOS scores (26). Publication bias was assessed through visual inspection of funnel plot symmetry and further evaluated using Egger's regression test (30). A two-sided *p* value < 0.05 was considered indicative of statistical significance. All analyses were performed using RevMan (version 5.3; Cochrane Collaboration, Oxford, UK) and Stata (version 17.0; StataCorp, College Station, TX, USA). Certainty of evidence for the primary outcome was evaluated using the Grading of Recommendations Assessment, Development and Evaluation (GRADE) framework (26). The certainty of evidence was assessed across the domains of risk of bias, inconsistency, indirectness, imprecision, and publication bias and categorized as high, moderate, low, or very low.

## Results

### Database search results

**Figure 1:**
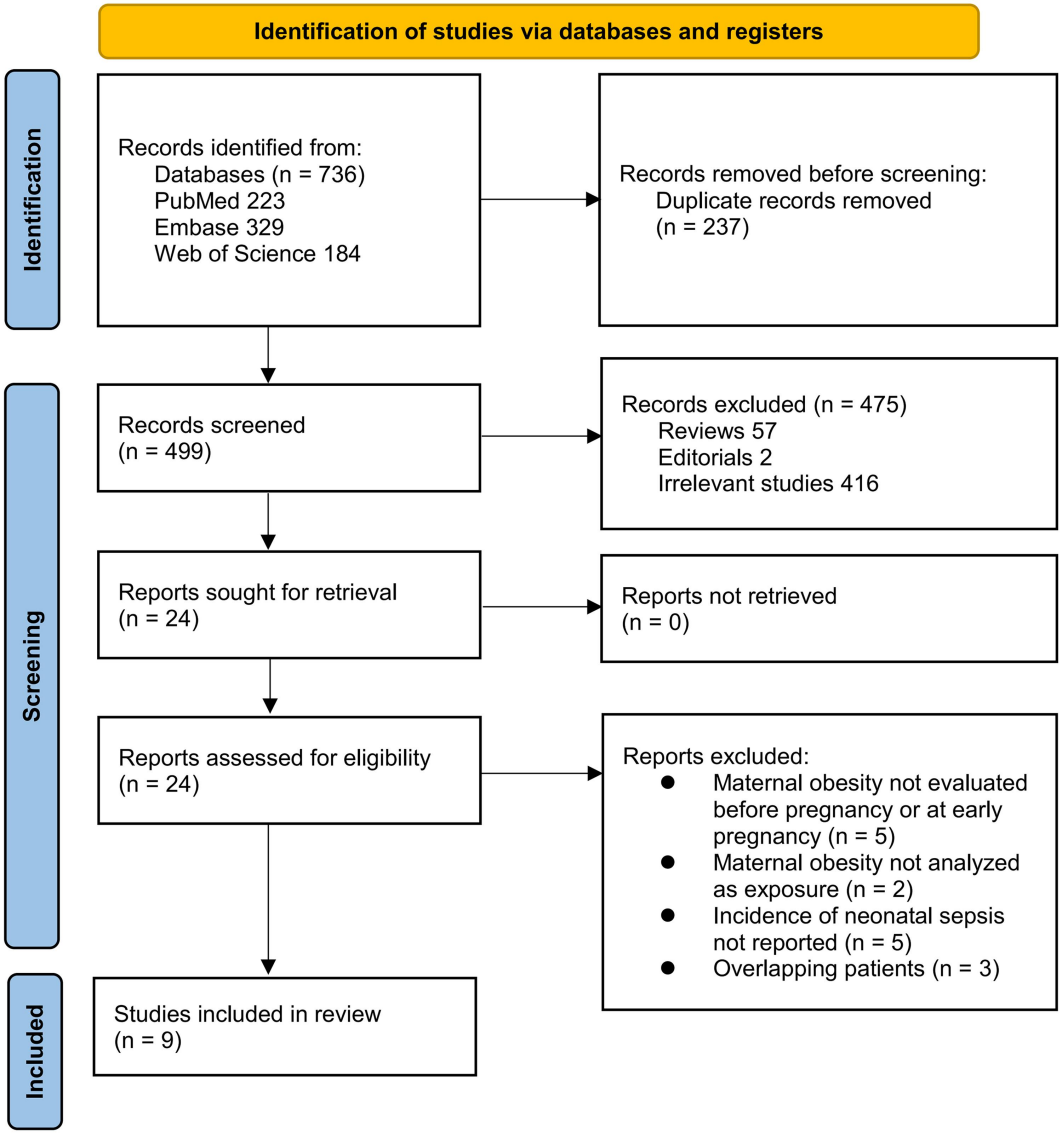
PRISMA 2020 flow diagram of the study identification, screening, eligibility assessment, and inclusion process for the meta-analysis.

The study selection process is illustrated in Figure 1. A total of 736 records were retrieved from the three databases, of which 237 duplicates were removed. Following title and abstract screening, 475 records were excluded for not meeting the predefined inclusion criteria. The full texts of 24 articles were subsequently evaluated independently by two reviewers, and 15 were excluded for the reasons detailed in Figure 1. Ultimately, nine studies met the eligibility criteria and were included in the quantitative meta-analysis (17–25).

### Overview of study characteristics

The main characteristics of the included studies are summarized in Table 1. A total of nine observational studies (17–25) involving 2,146,883 women were included in the meta-analysis. Among these, one study was prospective (17), while the remaining eight studies were retrospective cohort studies (18–25). The included studies were conducted across multiple geographic regions, including the United States (18, 19, 23, 25), Sweden (21), Germany (17), Canada (20), Turkey (22), and Bangladesh (24). The included populations comprised both high-risk women with preterm delivery (18, 20), and general obstetric cohorts involving both preterm and term delivery, such as those with fetal growth restriction (FGR) (23), diabetes (24), and hospital (17, 19, 22, 25) or population-based (21) general pregnant women. The mean maternal age ranged from 25.4 to 31.5 years, while the mean GA at delivery ranged from 29.2 to 39.0 weeks. Four studies evaluated maternal obesity exclusively using prepregnancy BMI (17–20), whereas the remaining studies additionally allowed BMI measured at the first prenatal visit or during early pregnancy (21–25). Maternal obesity was generally defined as BMI ≥30 kg/m^2^, although three studies further stratified obesity severity according to WHO obesity classes (19, 21, 25). Neonatal sepsis was diagnosed using various methods, including culture-confirmed sepsis in four studies (18, 20, 21, 25), and diagnosis from ICD-9 codes or medical chart diagnosis codes in five studies (17, 19, 22–24). Accordingly, 6,073 cases of neonatal sepsis were observed. Five studies (17–19, 21, 25) reported multivariable-adjusted estimates controlling for important confounding factors, including maternal age, GA, parity, smoking, birth weight, obstetric complications, and other maternal or neonatal characteristics, although the extent of adjustment varied substantially among studies. Only estimates from univariate analyses were reported in the other four studies (20, 22–24).

**Table 1:** Characteristics of the included studies.

Study	Design	Country	Source of women	No. of women included	Mean maternal age (years)	Mean GA at delivery (weeks)	Timing of BMI evaluation	Cutoff of BMI for maternal obesity (kg/m^2^)	No. of neonates born to women with obesity	Mean birth weight of neonates born to women with obesity (g)	Male sex of neonates (%)	Diagnostic methods for neonatal sepsis	No. of cases with neonatal sepsis	Variables matched or adjusted
Kalk (17)	P	Germany	Consecutive singleton deliveries at a single hospital	2,049	30	38.9	Pre-pregnancy	≥ 30	126	3,587	52.4	Medical chart diagnosis codes, overall sepsis	16	Maternal age, smoking, parity, child sex, and GA
Faucett (18)	R	USA	Women with PPROM pregnancies	1,288	NR	29.3	Pre-pregnancy	≥ 30	325	1,290	53.3	Culture-proven sepsis, overall sepsis	220	Magnesium sulfate, steroids, maternal diabetes, GA at delivery, indomethacin, birth weight, chorioamnionitis
Kim (19)	R	USA	Pregnant women without chronic diseases	112,309	27.5	NR	Pre-pregnancy	WHO categories: Class I (30–34.9), Class II (35–39.9), Class III (≥40)	19,482	NR	NR	ICD-9 codes, overall sepsis	2,673	Maternal age, race-ethnicity, parity, insurance, smoking, alcohol use, study site
Faden (20)	R	Canada	Women with preterm delivery in a single hospital	759	30.8	29.2	Pre-pregnancy	≥ 30	275	1,443	52.5	Clinical diagnosis with culture-proven sepsis, overall sepsis	94	None
Villamor (21)	R	Sweden	Nationwide population-based birth	1,971,346	30.1	39	Early pregnancy (first prenatal visit, median 10.3 weeks gestation)	WHO categories: Class I (30–34.9), Class II (35–39.9), Class III (≥40)	2,08,959	NR	51.4	Culture-confirmed bacterial sepsis, EOS	2,913	Maternal age, GA, country of origin, education level, cohabitation with partner, parity, height, smoking during pregnancy, year of delivery
Altin (22)	R	Turkey	Pregnant women undergoing cesarean section at a single hospital	151	31.5	38	Pre-pregnancy or first prenatal visit	≥30	110	3,321	NR	Medical chart diagnosis codes, EOS	4	None
Whelan (23)	R	USA	Singleton pregnancies complicated by FGR at a single hospital	379	25.4	37.3	Pre-pregnancy or first prenatal visit	≥ 30	64	2,316	NR	Medical chart diagnosis codes, overall sepsis	3	None
Jones (25)	R	USA	Singleton pregnancies delivering at a single hospital	58,497	28.2	39	First prenatal visit (median 9–10 weeks)	WHO categories: Class I (30–34.9), Class II (35–39.9), Class III (≥40)	21,276	3,411	NR	Culture-positive sepsis, overall sepsis	146	GA and parity
Ara (24)	R	Bangladesh	Diabetic pregnant women at a single hospital	105	31.2	37.6	Pre-pregnancy or first prenatal visit	≥ 30	52	3,510	NR	Medical chart diagnosis codes, overall sepsis	4	None

NR, not reported; P, prospective cohort study; R, retrospective cohort study; GA, gestational age; BMI, body mass index; PPROM, preterm premature rupture of membranes; WHO, World Health Organization; ICD-9, International Classification of Diseases, Ninth Revision; EOS, early-onset sepsis; FGR, fetal growth restriction.

### Study quality evaluation

The methodological quality of the included studies was assessed using the NOS, with detailed results summarized in Table 2. The NOS scores ranged from 6 to 9, indicating moderate to high overall methodological quality. One study (21) achieved the maximum NOS score of 9, reflecting excellent cohort representativeness, appropriate exposure ascertainment, adequate adjustment for GA and other confounders, reliable outcome assessment, and sufficient follow-up. Three studies (17, 18, 25) scored 8, indicating generally robust methodological quality with only minor limitations. One study (20) received a score of 7, mainly due to limited adjustment for confounding factors. Four studies (19, 22–24) scored 6, primarily because of insufficient adjustment for GA or other confounders, less rigorous outcome assessment methods, or inadequate representativeness of the exposed cohort. Overall, exposure ascertainment was generally appropriate across studies because maternal obesity was mainly evaluated using prepregnancy or early-pregnancy BMI. However, heterogeneity existed regarding study populations, adjustment strategies, and diagnostic methods for neonatal sepsis. Taken together, the included studies were considered to be of moderate to high quality, supporting the reliability of the pooled findings while acknowledging potential methodological heterogeneity among studies.

**Table 2:** Study quality evaluation via the Newcastle-Ottawa scale.

Study	Representativeness of the exposed cohort	Selection of the non-exposed cohort	Ascertainment of exposure	Outcome not present at baseline	Control for GA at delivery	Control for other confounding factors	Assessment of outcome	Enough long follow-up duration	Adequacy of follow-up of cohorts	Total
Kalk (17)	1	1	1	1	1	1	0	1	1	8
Faucett (18)	0	1	1	1	1	1	1	1	1	8
Kim (19)	0	1	1	1	0	1	0	1	1	6
Faden (20)	1	1	1	1	0	0	1	1	1	7
Villamor (21)	1	1	1	1	1	1	1	1	1	9
Altin (22)	1	1	1	1	0	0	0	1	1	6
Whelan (23)	1	1	1	1	0	0	0	1	1	6
Jones (24)	0	1	1	1	1	1	1	1	1	8
Ara (25)	1	1	1	1	0	0	0	1	1	6

### Results of the meta-analysis

**Figure 2:**
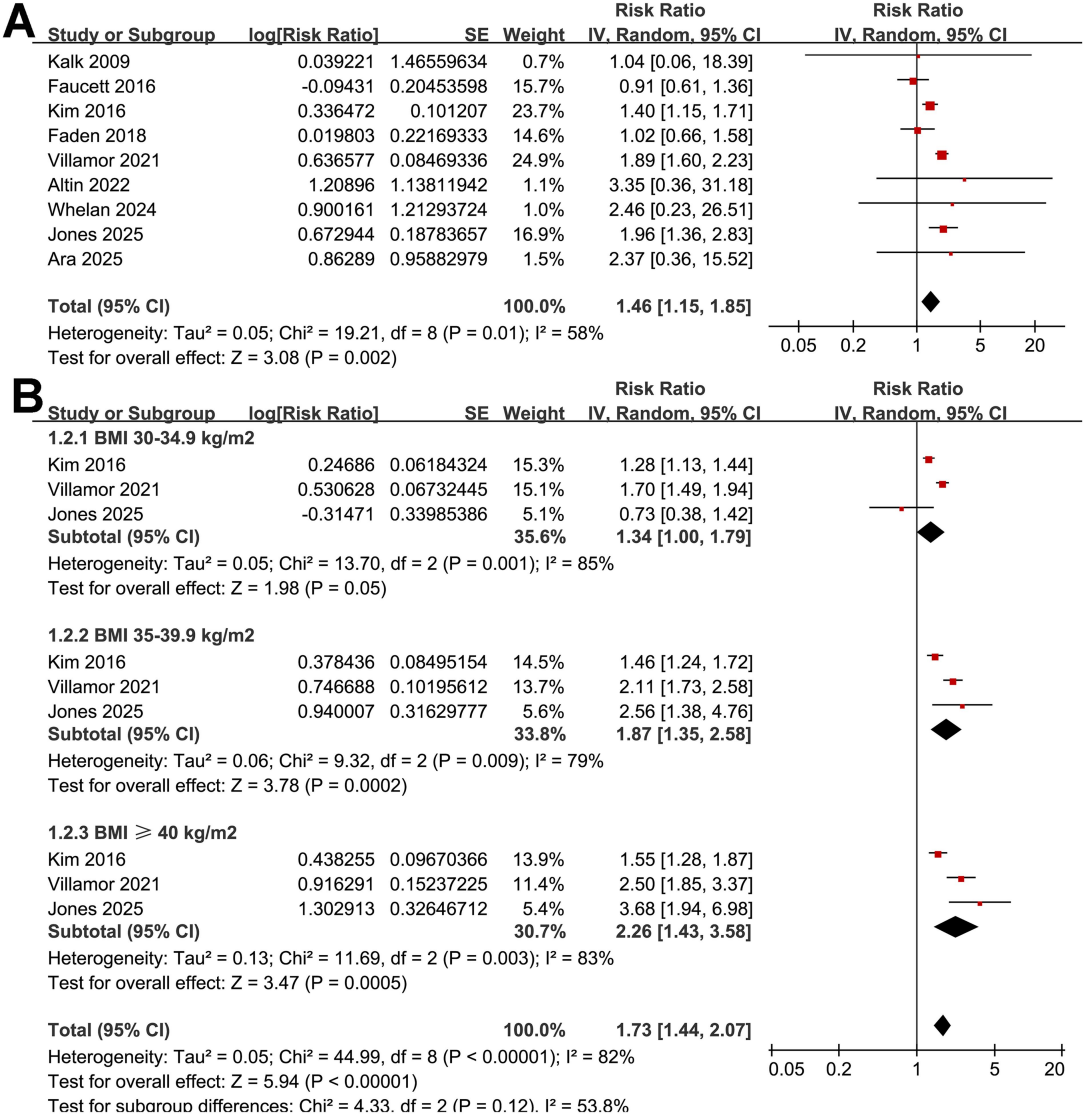
Forest plots showing the meta-analysis of the association between maternal obesity and risk of neonatal sepsis: **(A)** forest plots for the overall meta-analysis; and **(B)** forest plots for the stratified analysis according to BMI categories.

The pooled results of the nine studies (17–25) showed that maternal obesity before or during early pregnancy was associated with a higher risk of neonatal sepsis in offspring (RR: 1.46, 95% CI: 1.15 to 1.85, *p* = 0.002; Figure 2A) with moderate heterogeneity (*p* for Cochrane *Q* test = 0.01; I^2^ = 58%). Further sensitivity analysis by excluding one study at a time did not significantly change the results (RR: 1.33 to 1.60, all *p* < 0.05), indicating the robustness of the finding. Stratified analyses according to the severity of maternal obesity showed numerically increased RRs for neonatal sepsis among women with BMI values of 30–34.9, 35–39.9, and ≥ 40 kg/m^2^ (RRs = 1.34, 1.87, and 2.26, respectively), although the differences among the groups did not reach statistical significance (*p* = 0.12; Figure 2B).

### Results of the subgroup analyses

**Figure 3:**
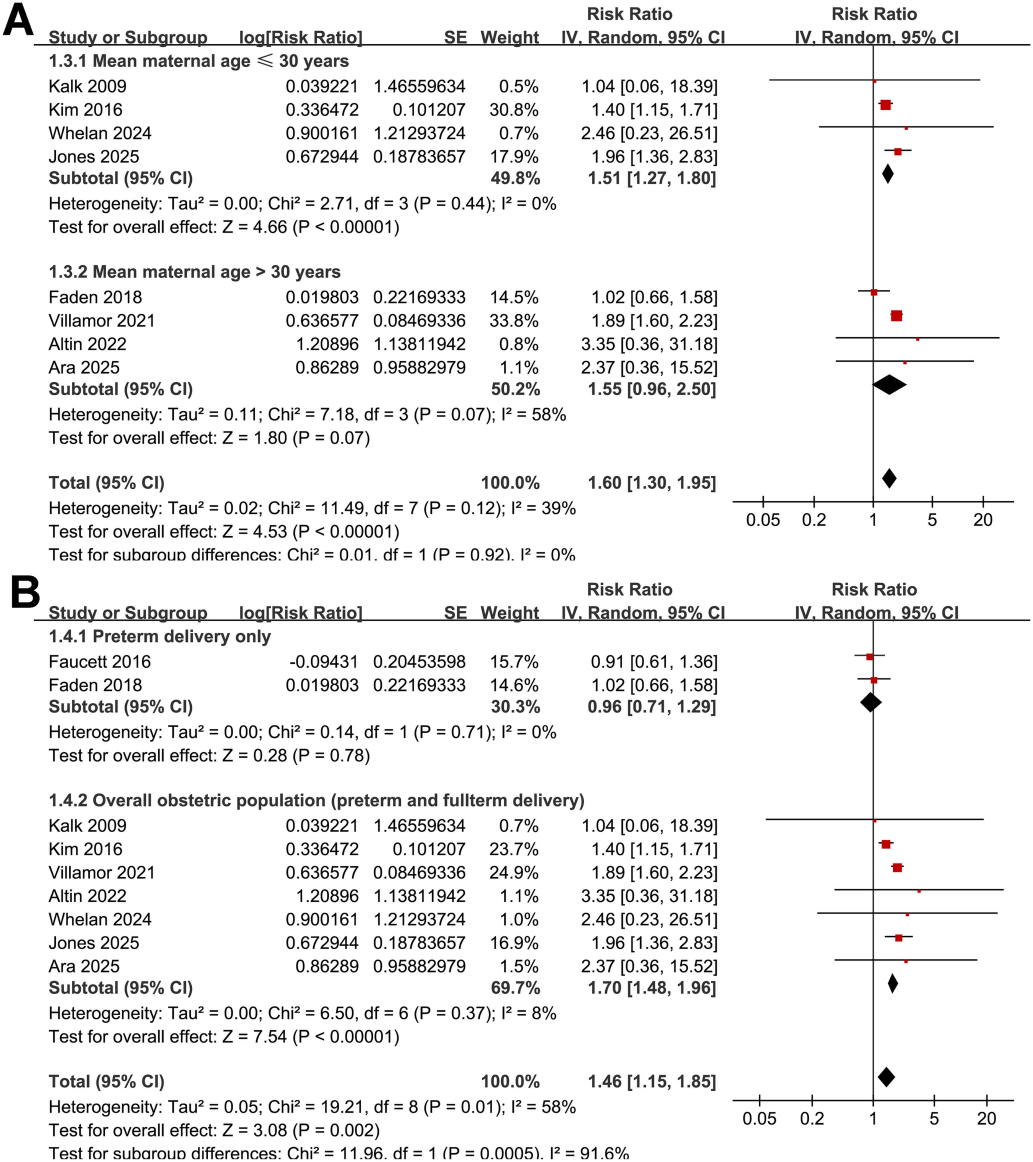
Forest plots showing the subgroup analysis of the association between maternal obesity and risk of neonatal sepsis: **(A)** forest plots for the subgroup analysis according to the maternal mean age; and **(B)** forest plots for the subgroup analysis according to the source of the population.

**Figure 4:**
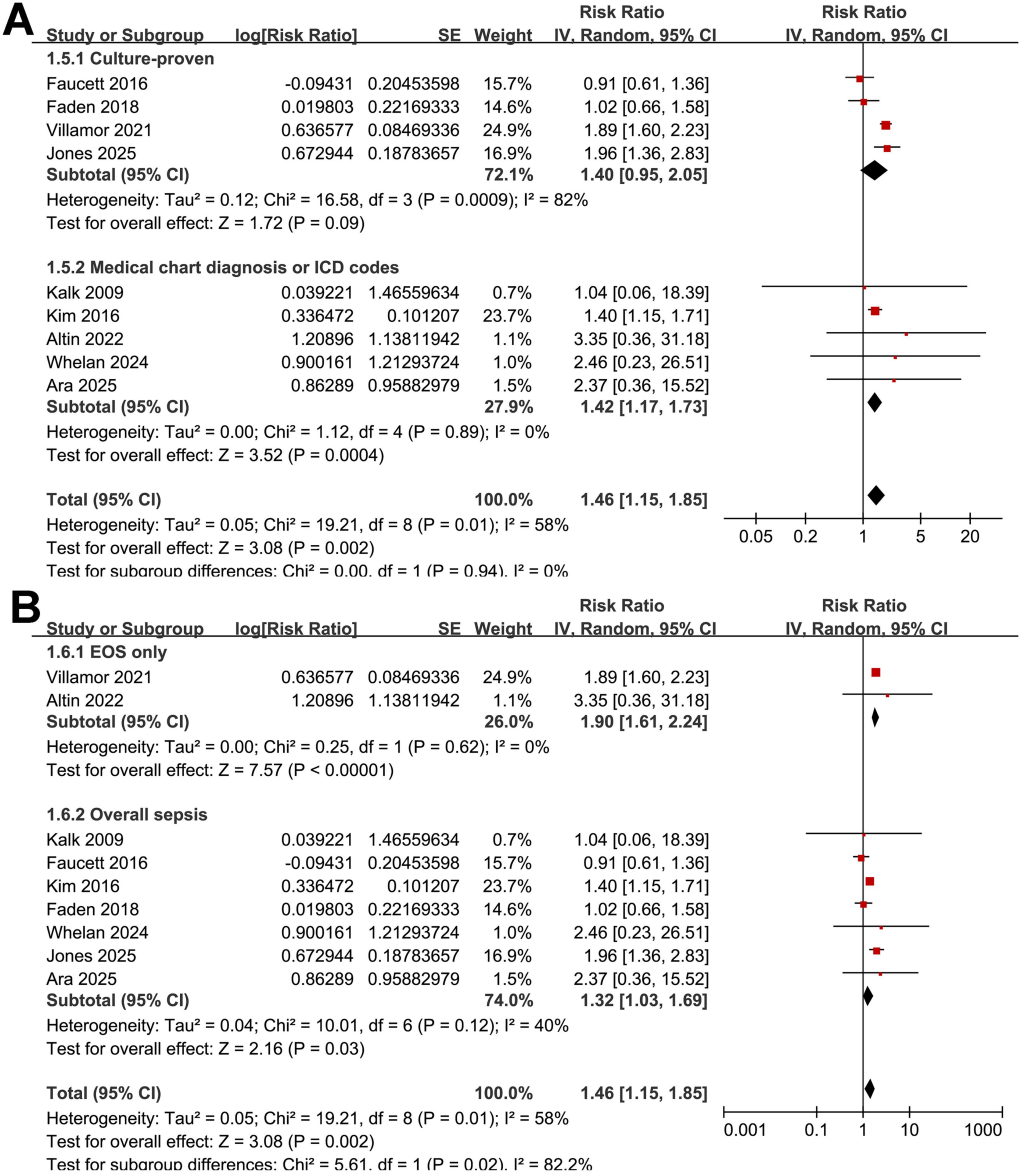
Forest plots showing the subgroup analysis of the association between maternal obesity and risk of neonatal sepsis: **(A)** forest plots for the subgroup analysis according to the methods for the validation of neonatal sepsis; and **(B)** forest plots for the subgroup analysis according to the definition of neonatal sepsis.

**Figure 5:**
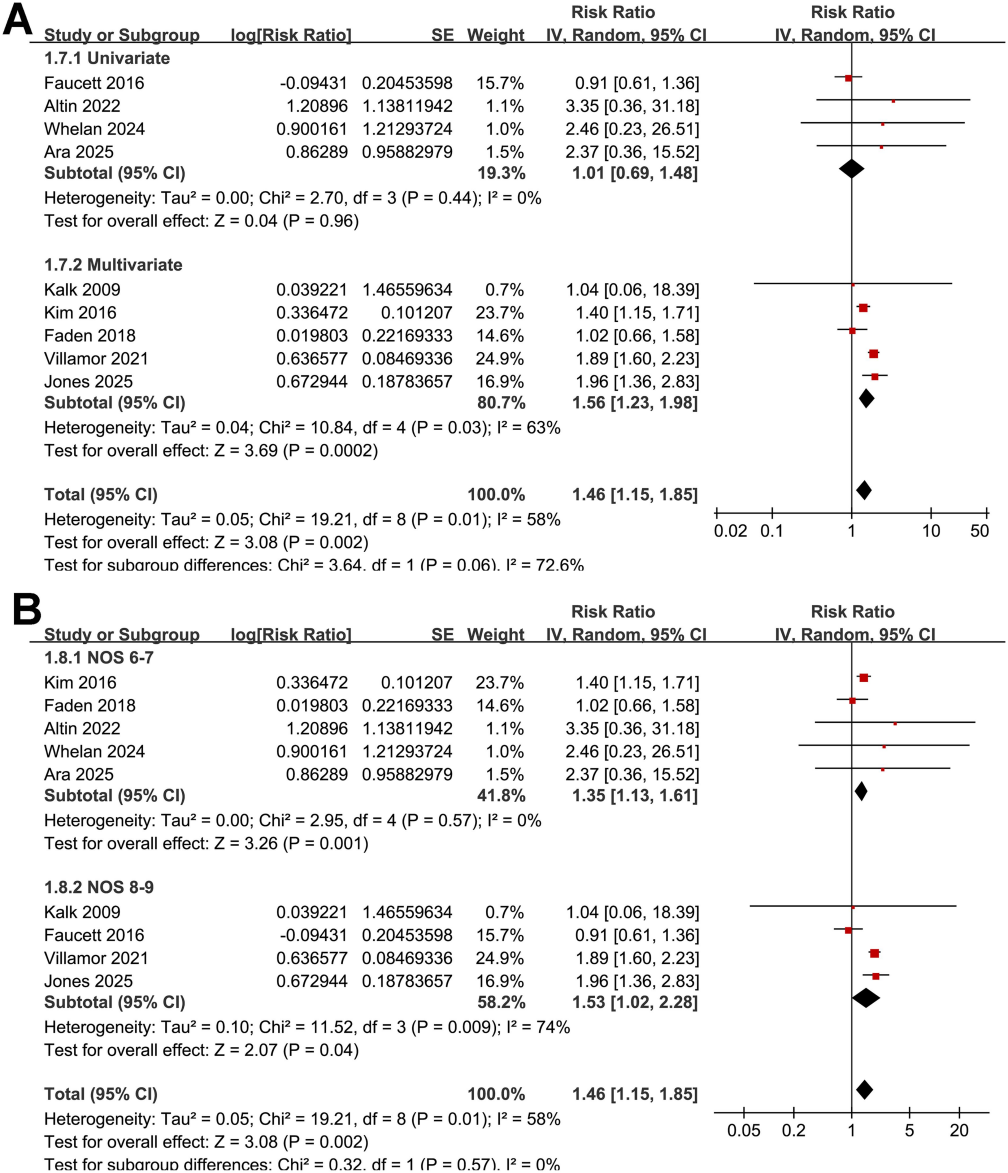
Forest plots showing the subgroup analysis of the association between maternal obesity and risk of neonatal sepsis: **(A)** forest plots for the subgroup analysis according to the analytic models; and **(B)** forest plots for the subgroup analysis according to the study quality scores.

Further subgroup analyses showed a consistent association between maternal obesity and the risk of neonatal sepsis in women with mean age ≤30 and >30 years (RR: 1.51 vs. 1.55, *p* for subgroup difference = 0.92; Figure 3A). However, a significant association between maternal obesity and the risk of neonatal sepsis was observed in studies with general obstetric populations including both preterm and term deliveries, but not in studies including only women with preterm delivery (RR: 1.70 vs. 0.96, *p* for subgroup difference <0.001; Figure 3B). Similar results were observed in studies with neonatal sepsis diagnosed by culture and ICD or medical chart codes (RR: 1.40 vs. 1.42, *p* for subgroup difference = 0.94; Figure 4A). However, a stronger association was observed for studies reporting EOS only as compared to those reporting overall neonatal sepsis (RR: 1.90 vs. 1.32, *p* for subgroup difference = 0.02; Figure 4B). The results were not significantly different between studies with univariate and multivariate analyses (*p* for subgroup difference = 0.06; Figure 5A) or between the studies with NOS of 6–7 and 8–9 (*p* for subgroup difference = 0.57; Figure 5B).

### Results of the meta-regression analysis

Univariate meta-regression analysis was performed to evaluate the potential influence of study characteristics on the association between maternal obesity and neonatal sepsis, as summarized in Table 3. The results showed that mean GA at delivery and mean birthweight of neonates born to women with obesity were significantly associated with the pooled effect size. Specifically, studies with higher mean GA at delivery and higher neonatal birthweight tended to show stronger associations between maternal obesity and neonatal sepsis. However, these findings should be interpreted cautiously because the meta-regression analyses were based on only nine studies. Consequently, the statistical power was limited, the adjusted R² estimates may be unstable, and the results are susceptible to ecological bias. Therefore, these findings should be considered exploratory and hypothesis-generating rather than definitive explanations of between-study heterogeneity. In contrast, mean maternal age and NOS scores were not significantly associated with the pooled results (both *p* > 0.05).

**Table 3:** Results of univariate meta-regression analysis.

Variables	RR for the association maternal obesity and the risk of neonatal sepsis			
	Coefficient	95% CI	*p* values	Adjusted R^2^
Mean maternal age (years)	0.0084	−0.2561 to 0.2730	0.94	0%
Mean GA at delivery (Weeks)	0.070	0.028 to 0.113	0.007	100%
Mean birthweight of neonates born to women with obesity (g)	0.00035	0.00006 to 0.00065	0.03	100%
NOS	0.054	−0.263 to 0.370	0.70	0%

RR, relative risk; CI, confidence interval; GA, gestational age; NOS, Newcastle–Ottawa Scale;.

### Publication bias

**Figure 6:**
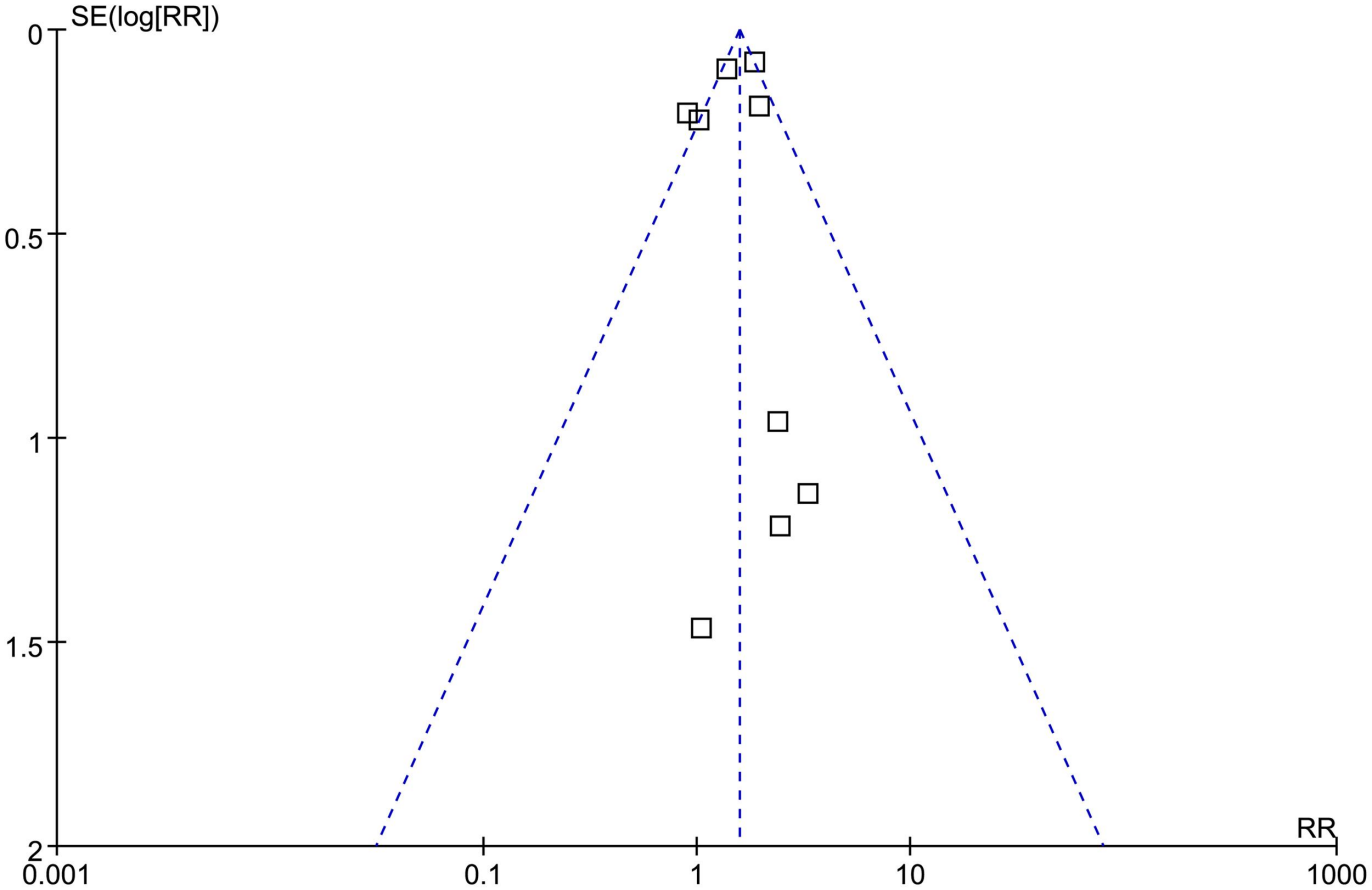
Funnel plots evaluating the publication bias for the meta-analysis of the association between maternal obesity and risk of neonatal sepsis.

As illustrated in Figure 6, the funnel plots assessing the association between maternal obesity and the risk of neonatal sepsis appeared generally symmetrical. In line with this observation, Egger's regression test did not detect significant publication bias (*p* = 0.54). Nevertheless, these findings should be interpreted with caution due to the relatively small number of included studies (*k* = 9).

#### Certainty of evidence

The certainty of evidence for the association between maternal obesity and neonatal sepsis was evaluated using the GRADE framework (Table 4). The overall certainty of evidence was rated as low. Although moderate statistical heterogeneity was observed (I^2^ = 58%), inconsistency was not considered serious because prespecified subgroup analyses and meta-regression identified clinically plausible sources of heterogeneity, including differences in study populations, gestational age at delivery, birthweight, and sepsis definitions. The certainty rating was primarily limited by the observational nature of the included studies and the possibility of residual confounding.

**Table 4:** GRADE assessment of the certainty of evidence for the association between maternal obesity and the risk of neonatal sepsis.

Outcome	No. of studies (cohorts)	Participants	Study design	Risk of bias	Inconsistency	Indirectness	Imprecision	Publication bias	Effect estimate/Certainty
Neonatal sepsis	9 studies	2,146,883	Observational cohort studies	Serious^a^	Not serious^b^	Not serious	Not serious	Not detected^c^	RR 1.46 (95% CI 1.15–1.85) Low ⊕⊕◯◯

Explanations.

a The evidence was derived exclusively from observational cohort studies. Although several studies adjusted for important confounders, four studies reported only univariate estimates and residual confounding cannot be completely excluded.

b Moderate between-study heterogeneity was observed (I^2^ = 58%). However, prespecified subgroup analyses and meta-regression identified clinically plausible sources of heterogeneity, including differences in study populations, gestational age at delivery, birthweight, and sepsis definitions. The direction of association remained generally consistent across analyses.

c Funnel plot inspection and Egger's regression test did not indicate significant publication bias.

## Discussion

This meta-analysis of nine cohort studies involving more than two million women suggests that maternal obesity before or during early pregnancy is associated with an increased risk of neonatal sepsis. The association remained generally robust across sensitivity and subgroup analyses and may be more evident in certain clinical settings, supporting a potential role of maternal obesity as an important maternal factor associated with neonatal infectious vulnerability.

Several pathophysiological mechanisms may explain the observed association between maternal obesity and neonatal sepsis. Maternal obesity is characterized by chronic low-grade systemic inflammation, which is associated with increased circulating levels of proinflammatory cytokines, including tumor necrosis factor-α, interleukin-6, and C-reactive protein (31, 32). Obesity-related inflammatory activation may impair placental function, alter maternal-fetal immune interactions, and contribute to dysregulated fetal immune development (33). In addition, maternal obesity has been associated with altered gut and vaginal microbiota, which may influence neonatal microbial colonization during delivery and early life (34, 35). Experimental studies have also suggested that maternal obesity may impair neonatal innate immune responses, including neutrophil function and cytokine regulation, potentially increasing susceptibility to bacterial infection after birth (36, 37). Furthermore, maternal obesity is strongly associated with multiple adverse obstetric conditions that are themselves related to neonatal sepsis, including gestational diabetes, hypertensive disorders of pregnancy, cesarean delivery, prolonged labor, fetal macrosomia, and neonatal intensive care unit admission (11, 12). Obesity-related metabolic dysfunction and placental inflammation may additionally increase the likelihood of intrauterine inflammation or chorioamnionitis, which could predispose neonates to EOS (38–40). Therefore, both direct inflammatory-immunological mechanisms and indirect obstetric complications may contribute to the increased risk of neonatal sepsis observed among offspring of women with obesity.

Interestingly, subgroup analyses showed that the association between maternal obesity and neonatal sepsis was significant in studies involving general obstetric populations but not in studies restricted to women with preterm delivery. This finding may be explained by the markedly elevated baseline risk of neonatal sepsis among preterm infants, in whom prematurity-related factors such as immature immune function, prolonged hospitalization, invasive procedures, respiratory support, and central venous catheter use may dominate the pathogenesis of neonatal infection (41). Under these conditions, the relative contribution of maternal obesity to neonatal sepsis risk may become attenuated or more difficult to detect. This interpretation is further supported by the meta-regression findings, which showed that studies with higher mean GA at delivery and higher neonatal birthweight tended to demonstrate stronger associations between maternal obesity and neonatal sepsis. Since lower GA and lower birthweight are among the strongest independent risk factors for neonatal sepsis, the influence of maternal obesity may be relatively overshadowed in populations with substantial prematurity-related risk burden. In contrast, in general obstetric populations with lower baseline infectious risk, obesity-related inflammatory and metabolic effects may become more clinically apparent.

Another noteworthy finding was that the association appeared stronger for EOS than for overall neonatal sepsis. EOS is closely related to maternal and perinatal conditions, including maternal genital tract colonization, intrauterine infection, placental inflammation, prolonged rupture of membranes, and delivery-related microbial exposure (42). Therefore, maternal obesity may exert a more direct influence on EOS through maternal inflammatory activation, altered maternal microbiota, and obesity-associated obstetric complications occurring during pregnancy and delivery. In contrast, late-onset neonatal sepsis is more strongly influenced by postnatal environmental factors, prolonged hospitalization, invasive procedures, and NICU-related exposures, which may dilute the relative contribution of maternal obesity (43). These findings support the biological plausibility of the observed association between maternal obesity and neonatal sepsis, particularly for EOS.

This meta-analysis has several strengths. First, an up-to-date and comprehensive literature search was performed across multiple databases, enabling inclusion of the currently available evidence regarding maternal obesity and neonatal sepsis. Second, all included studies were cohort studies with longitudinal follow-up, which strengthens the temporal relationship between maternal obesity and subsequent neonatal sepsis. Third, maternal obesity was primarily defined using prepregnancy or early-pregnancy BMI, which is more reliable and clinically meaningful than BMI measured later during pregnancy because it is less affected by gestational weight gain (44). Fourth, multiple predefined subgroup analyses, sensitivity analyses, and meta-regression analyses were performed to explore the robustness of the findings and potential sources of heterogeneity. In addition, studies involving different obesity severity categories were separately analyzed, allowing exploration of a possible dose-dependent association.

Several limitations of this meta-analysis should also be acknowledged. First, most included studies were retrospective observational studies, which may be susceptible to selection bias, recall bias, and residual confounding (45). Second, heterogeneity existed among studies regarding maternal demographic characteristics, source populations, definitions of neonatal sepsis, and methods for outcome ascertainment. Specifically, neonatal sepsis was identified using either culture-confirmed diagnoses or ICD/medical chart-based diagnoses, and the included populations ranged from general obstetric cohorts to women with preterm delivery, fetal growth restriction, or diabetes. Differences in adjustment strategies, geographic locations, healthcare systems, and clinical practices may have also contributed to the observed heterogeneity. Although subgroup analyses and meta-regression were performed to explore potential sources of variability, these approaches have inherent limitations. In particular, the meta-regression findings suggesting that GA and birthweight explained a substantial proportion of between-study heterogeneity should be interpreted cautiously because only nine studies were available for analysis. Such study-level analyses may be unstable and are susceptible to ecological bias; therefore, these findings should be regarded as exploratory and hypothesis-generating rather than definitive explanations of heterogeneity. In addition, although maternal obesity was consistently defined as BMI ≥ 30 kg/m² across the included studies, some studies further classified obesity according to WHO obesity subclasses, which may have contributed to differences in effect estimates. Nevertheless, subgroup analyses according to obesity severity showed a generally consistent pattern of increasing risk with higher BMI categories. More importantly, the timing of BMI assessment varied across studies, with some using prepregnancy BMI and others using BMI measured at the first prenatal visit or during early pregnancy. Although early-pregnancy BMI is widely accepted as a proxy for prepregnancy BMI and is less influenced by gestational weight gain than later pregnancy measurements, differences in measurement timing may have resulted in some exposure misclassification and reduced comparability across studies. Third, adjustment strategies differed substantially among studies. Four studies reported only univariate estimates, whereas five studies provided multivariable-adjusted estimates. Pooling adjusted and unadjusted effect estimates may introduce bias because unadjusted analyses are more susceptible to confounding. Although subgroup analysis according to analytic model showed no significant subgroup difference, residual confounding cannot be excluded. In addition, several studies did not adequately control for important factors such as GA at delivery and other maternal or neonatal characteristics, which may have influenced the observed associations. Fourth, publication bias could not be completely excluded despite the absence of significant asymmetry in funnel plot analysis and Egger's test because the number of included studies was relatively small. Finally, as with all meta-analyses of observational studies, the current findings demonstrate an association rather than a causal relationship between maternal obesity and neonatal sepsis.

The findings of this meta-analysis may have important clinical implications. Maternal obesity before or during early pregnancy may represent a potentially modifiable maternal factor associated with neonatal sepsis risk. These findings highlight the importance of preconception counseling, weight management, and optimized antenatal care in women with obesity. Clinicians may also consider closer neonatal monitoring for infection among offspring of women with obesity, particularly in populations at risk for EOS. However, given the observational nature of the included studies and the heterogeneity among studies, the results should be interpreted cautiously. Future prospective large-scale cohort studies with standardized definitions of maternal obesity and neonatal sepsis are needed to validate these findings. Studies incorporating detailed maternal metabolic, inflammatory, microbiological, and placental biomarkers may further clarify the biological mechanisms linking maternal obesity to neonatal sepsis. In addition, individual participant data meta-analyses may help determine whether the association differs according to GA, neonatal birthweight, delivery mode, maternal metabolic comorbidities, and obesity severity.

## Conclusions

In conclusion, maternal obesity before or during early pregnancy was associated with an increased risk of neonatal sepsis in offspring. The association appeared stronger in general obstetric populations, among neonates with higher GA and birthweight, and for EOS. These findings suggest that the influence of maternal obesity on neonatal infectious risk may be more evident in populations where the substantial baseline risk associated with prematurity is less dominant. Nevertheless, further well-designed prospective studies are warranted to confirm these findings and clarify the underlying mechanisms.

## Data Availability

The original contributions presented in the study are included in the article/Supplementary Material, further inquiries can be directed to the corresponding author/s.
