# Supplementary material for: Maternal obesity and the risk of neonatal sepsis: a meta-analysis
Source: Front Pediatr. 2026 Jul 31;14:1886925. doi: 10.3389/fped.2026.1886925 (PMC13473157; doi:10.3389/fped.2026.1886925)
Supplement: Supplementary file 1 [file Table1.docx]

**Detailed search strategy for each database**

**PubMed**

("Obesity"[Mesh] OR "Overweight"[Mesh] OR obes*[tiab] OR overweight[tiab] OR "body mass index"[tiab] OR BMI[tiab] OR "prepregnancy BMI"[tiab] OR "pre-pregnancy BMI"[tiab] OR "maternal obesity"[tiab]) AND ("Sepsis"[Mesh] OR sepsis[tiab] OR septicemia[tiab] OR "bloodstream infection"[tiab] OR "neonatal infection"[tiab]) AND ("Infant, Newborn"[Mesh] OR neonat*[tiab] OR newborn*[tiab] OR "early-onset sepsis"[tiab] OR "late-onset sepsis"[tiab])

**Embase**

('obesity'/exp OR 'overweight'/exp OR obes*:ti,ab OR overweight:ti,ab OR 'body mass index':ti,ab OR BMI:ti,ab OR 'maternal obesity':ti,ab OR 'prepregnancy bmi':ti,ab) AND ('sepsis'/exp OR sepsis:ti,ab OR septicemia:ti,ab OR 'bloodstream infection':ti,ab OR 'neonatal infection':ti,ab) AND ('newborn'/exp OR neonat*:ti,ab OR newborn*:ti,ab OR 'early onset sepsis':ti,ab OR 'late onset sepsis':ti,ab)

**Web of Science**

TS = ((obes* OR overweight OR "body mass index" OR BMI OR "maternal obesity" OR "prepregnancy BMI" OR "pre-pregnancy BMI") AND (sepsis OR septicemia OR "bloodstream infection" OR "neonatal infection") AND (neonat* OR newborn* OR "early-onset sepsis" OR "late-onset sepsis"))
